# Chromatin and noncoding RNA-mediated mechanisms of gastric tumorigenesis

**DOI:** 10.1038/s12276-023-00926-0

**Published:** 2023-01-19

**Authors:** Adrian Kwan Ho Loe, Lexin Zhu, Tae-Hee Kim

**Affiliations:** 1 Program in Developmental & Stem Cell Biology, The Hospital for Sick Children, Toronto, ON M5G 0A4 Canada; 2 Department of Molecular Genetics, University of Toronto, Toronto, ON M5S 1A8 Canada

**Keywords:** Gastric cancer, Gastric cancer

## Abstract

Gastric cancer (GC) is one of the most common and deadly cancers in the world. It is a multifactorial disease highly influenced by environmental factors, which include radiation, smoking, diet, and infectious pathogens. Accumulating evidence suggests that epigenetic regulators are frequently altered in GC, playing critical roles in gastric tumorigenesis. Epigenetic regulation involves DNA methylation, histone modification, and noncoding RNAs. While it is known that environmental factors cause widespread alterations in DNA methylation, promoting carcinogenesis, the chromatin- and noncoding RNA-mediated mechanisms of gastric tumorigenesis are still poorly understood. In this review, we focus on discussing recent discoveries addressing the roles of histone modifiers and noncoding RNAs and the mechanisms of their interactions in gastric tumorigenesis. A better understanding of epigenetic regulation would likely facilitate the development of novel therapeutic approaches targeting specific epigenetic regulators in GC.

## Gastric cancer: Interplay of epigenetic regulators

Detailed studies of epigenetic regulation in gastric cancer could provide novel targets for combination therapies. Gastric cancer development is heavily influenced by environmental factors such as diet, which drive epigenetic changes to DNA and gene expression. Tae-Hee Kim and colleagues at the Hospital for Sick Children in Toronto, Canada, reviewed current understanding of epigenetic regulation in gastric cancer, focusing on non-coding RNA and modifications to histones, proteins that bind to DNA. Histone modifications alter chromatin, the DNA and protein complexes that form chromosomes, and this directly influences gene expression. These modifications are reversible, involving specific ‘writer’ and ‘eraser’ proteins. In gastric cancer, dysregulation of writer and eraser proteins alters the expression of cancer-related genes, while non-coding RNAs regulate critical cancer-promoting pathways. Further investigations are needed into how non-coding RNAs and histone modifications interact.

## Introduction

Gastric cancer (GC) is the fifth most common cancer and the fourth deadliest cancer in the world^1^. GC shows distinct incidence patterns across geographical regions: most gastric cancer cases are found in Eastern Asia and Europe, while it is the most common cancer in some of Middle Eastern countries. Although the overall incidence and mortality have decreased in most countries, likely due to better prevention and improved food preservation, increasing incidence rates among young adult populations have been observed in some countries, such as the UK and Sweden^2^. Notably, the treatment of advanced or metastatic GC, with a median overall survival of less than one year, has been extremely challenging^3^. Therefore, a better understanding of the disease mechanisms would likely help design more effective therapies.

Consistent with its distinct geographical distribution patterns, GC is a highly multifactorial disease regulated by genetic, epigenetic, and environmental factors. Common genetic factors include mutations in oncogenes and tumor suppressor genes involved in cancer initiation and progression^4^. The Cancer Genome Atlas (TCGA) project has identified common mutations and categorized GC into four major subtypes: Epstein‒Barr virus (EBV)-positive, microsatellite instability (MSI), genomically stable (GS), and chromosomal instability (CIN)^5^. Epigenetic factors are involved in the regulation of gene expression via mechanisms unrelated to the genetic sequence, which include DNA methylation, histone modification, and noncoding RNAs (ncRNAs)^6^. Interestingly, analysis of tumor suppressors and oncogenes has shown that changes in DNA methylation are more common than genetic mutations in GC, highlighting the importance of epigenetic regulation in gastric tumorigenesis^7^. Corroborating these data, another study showed that the impact of DNA methylation outweighs that of genetic mutations in GC when compared to esophageal cancer^8^. Similarly, environmental factors, most notably diet and infection, play major roles in gastric carcinogenesis. In fact, GC is strongly associated with two infectious agents categorized as type I carcinogens, *H. pylori* and EBV^9–13^.

Environmental and epigenetic factors are interconnected, as both diet and infection have been found to influence DNA methylation^14,15^. While DNA methylation in GC has been extensively reviewed^6,16–18^, other types of epigenetic regulation in GC are still poorly understood. As newly accumulating evidence suggests the critical roles of histone modification and ncRNAs in GC tumorigenesis, we discuss recent studies demonstrating their roles and mechanistic interactions.

## Histone modification and the chromatin landscape in gastric cancer

DNA is wrapped around octomeric histone cores to form nucleosomes, which are organized into chromatin. The organization of chromatin into accessible/active euchromatin or condensed/repressed heterochromatin directly influences gene expression, and it is tightly regulated by covalent modifications of the histone tails via methylation, acetylation and phosphorylation^19^. These modifications are reversible and are catalyzed by specialized “writer” and “eraser” proteins. For example, writers such as histone methyltransferases (HMTs) and histone acetyltransferases (HATs) deposit methyl and acetyl marks on histone tails, respectively. In contrast, erasers such as histone demethylases and histone deacetylases (HDACs) remove the respective marks^20^. Other types of modifications, such as ubiquitination, sumoylation, and GlcNAcylation for histones, are also possible, but they are much less studied. Writers and erasers also have specificity for the histone marks they regulate. For example, the catalytic subunit of the polycomb repressive complex 2 (PRC2), EZH2, catalyzes the methylation of histone H3 lysine 27 (H3K27), while H3K4 methylation is catalyzed by the complex proteins associated with the Set1 (COMPASS) complex. Similarly, histone lysine demethylase (KDM)6A/B/C removes methylation on H3K27, while LSD1/KDM1A demethylates H3K4me^20^.

The combination of histone modifications represents a code correlated with functional elements on the chromatin. For example, H3K4 methylation is associated with active elements: H3K4me3 and H3K4me1 are associated with enhancers and promoters, respectively^21,22^. Histone modifications can influence gene expression. For example, H3K27ac and H3K9ac are associated with active gene expression, while H3K27me3 is associated with transcriptional repression. The specific methylation state of the same amino acid residue can also function differently: H3K9me1 is associated with active promoters, while H3K9me2/3 is associated with promoter repression^23^.

Once histone marks are established, they can be recognized by “readers”, which are proteins that can interact with specific histone marks and exert a myriad of effects^20^. In this review, we will focus on ATPase-dependent chromatin remodeling complexes, which contain reader subunits and can directly influence the chromatin state by modifying either accessibility or nucleosome composition^24,25^.

### Alterations of histone modifiers establish the histone code underlying gastric carcinogenesis

Together, writer and eraser proteins establish the histone code, influencing the chromatin landscape and regulating a multitude of signaling pathways and genes. Unsurprisingly, many of these proteins play important roles in oncogenesis and tumor suppression. Since dysregulation of writer and eraser proteins in GC has been previously discussed^17,26,27^, here, we focus on the recent discoveries addressing their roles in GC.

#### Histone methyltransferases

Analysis of TCGA data showed that a number of histone modifiers are differentially expressed between GC and noncancer samples^28^. A more recent analysis of TCGA data focusing on HMTs found that the increased expression of *G9a*/*EHMT2*, an H3K9 methyltransferase, is associated with poor prognosis, while its oncogenic effect may be mediated by activating the expression of *MTOR* via increasing promoter H3K9me1^29–31^.

*PRC2*, which catalyzes H3K27me3, is also overexpressed in GC, and this overexpression is associated with poor prognosis^32^. Recent studies have demonstrated that PRC2 may promote GC development via multiple pathways. Xing et al. found that EZH2 represses *EBF1* expression by increasing promoter H3K27me, which subsequently activates the expression of *TERT*^33^, while another study showed that EZH2 may promote cancer via the downregulation of *PTEN*^34^. EZH2 was also found to induce H3K27me3 at the promoter of *P21*, a cell cycle regulator, and downregulate its expression^35^. In addition, EZH2 repressed the expression of *ERRγ*, a nuclear hormone receptor and a transcription factor, via H3K27me3, which led to the activation of an oncogene, *FOXM1*, in GC cells^36^.

Other HMTs have also been studied in recent years. For example, *WDR5* is a subunit of the KMT2/MLL complex that catalyzes H3K4me1 and H3K4me2, and its abnormal expression is associated with poor prognosis. Mechanistically, it increased H3K4me3 at the promoter of *CYCLIN D1*, a cell cycle gene frequently upregulated in human cancer, and activated its expression^37^. *KMT2* family genes were found to be mutated in approximately 10% of gastric cancer cases, and they were associated with PD-L1 expression, suggesting their potential roles in immune checkpoint therapy^38^. *SETD1A*, another HMT for H3K4, was overexpressed and localized to the promoter of *SNAIL*, a snail family transcriptional repressor, enhancing epithelial–mesenchymal transition (EMT) in GC cells^39^. In addition, the increased expression levels of *DOT1L*, an H3K79 methyltransferase, and protein arginine methyltransferases, such as *PRM5* and *PRMT6*, were also associated with poor prognosis^40–42^. Together, these recent studies demonstrate that writer proteins regulate histone modification across the GC genome and that their increased expression likely leads to the dysregulation of multiple genes and signaling pathways, promoting cancer development.

#### Histone demethylases

LSD1, the first histone demethylase identified, has been shown to act on both H3K4 and H3K9, thus having the potential to both repress and activate target gene expression. *LSD1* was found to be more highly expressed in GC than in normal tissue, and its increased expression was associated with metastasis and advanced cancer stages^43,44^. Mechanistically, it may promote EMT through the demethylation of promoter H3K4me2 and the subsequent downregulation of *CDH1* (*E-cadherin*) expression^44^.

#### Histone deacetylases, acetyltransferases, and kinases

When the expression levels of histone deacetylases and kinases were analyzed, the upregulation of *HDAC2*, *NEK6,* and *AURKA* was identified in GC^45,46^. Corroborating these findings, a recent study showed that HDAC2 may mediate the deacetylation of H3K9 at the *P21* promoter, leading to increased proliferation in GC cells^47^. In addition, the expression of the HATs *P300* and *CBP* was found to be upregulated in GC cells^48^. Together, these recent studies have shown that altered expression levels of histone modifiers are associated with gastric tumorigenesis, leading to cancer progression through the regulation of oncogene and tumor suppressor gene expression.

**Fig. 1:**
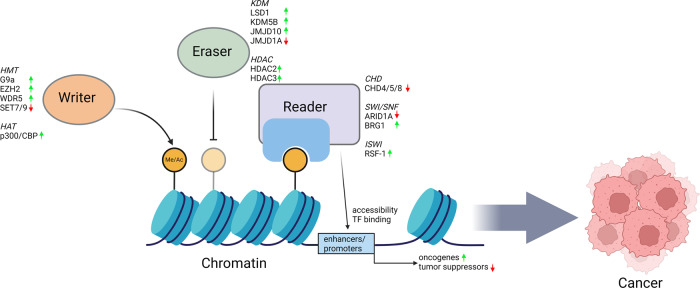
Dysregulation of histone modification during gastric carcinogenesis. Writer and eraser proteins modify the histone tail by adding or removing modifications such as methylation (Me) or acetylation (Ac). These modifications are recognized by reader proteins, which are found in chromatin remodeling complexes capable of altering chromatin accessibility and allowing the binding of transcription factors to regulate gene expression. In GC, the expression of these proteins is dysregulated, leading to the up- and downregulation of oncogenes and tumor suppressors.

Writers, erasers, and readers play a critical role in maintaining the proper chromatin state and gene expression for normal homeostasis. Mutations or altered expression levels of writers, erasers, and readers can lead to dysregulation of key signaling pathways and/or genes involved in gastric tumorigenesis (Fig. 1). The chromatin landscape, as defined by histone marks, is significantly remodeled during carcinogenesis. Therefore, new studies may benefit from genomic analyses of chromatin marks that are altered by the gain or loss of chromatin modifying factors. In addition, as most recent studies have utilized cancer cell lines, in vivo animal models or organs-on-a-chip combined with intact niche components would likely provide important, new insight into tumor heterogeneity and the microenvironment.

### Alterations in chromatin remodeling complexes play functional roles in gastric carcinogenesis

One way in which the histone code can direct functional rearrangement of chromatin is via the recruitment of chromatin remodeling complexes. Currently, as defined by their core ATPase subunits, four subfamilies of chromatin remodeling complexes exist: SWI/SNF, INO80, CHD, and ISWI^25^. Of note, in each of these complexes, genetic alterations and changes in gene expression have been linked to GC development.

#### CHD

The chromodomain helicase DNA-binding (CHD) subfamily of chromatin remodeling factors is so named because of the presence of the chromodomains that interact with methylated histones. The expression of *CHDs* has been examined in human GC cases. Notably, mutations and reduced expression of *CHD4* and *CHD8* were found in microsatellite instability (MSI)-high GC^49^. Consistently, knockdown of *CHD8* showed increased proliferation of GC cells^50^. The reduced expression of *CHD5* also correlated with poor prognosis and invasion^51^. CHDs can interact with other epigenetic regulators, such as MBDs and MTAs, to form the NuRD complex, and subunits of this complex are involved in GC development. *MBD2* was found to be downregulated in gastric preneoplastic lesions and tumors^52^. In contrast, the increased expression levels of *MTA1* and *MTA2* were associated with GC invasion and metastasis^53–55^. Indeed, the overexpression of *MTA2* promoted colony formation in GC cells, potentially via the upregulation of IL-11^56^. Interestingly, increased expression of *MTA3* was also found in GC compared to normal tissue. These studies highlight the importance of the subunit composition of the NuRD complex in gastric tumorigenesis^57^.

#### SWI/SNF

Unlike CHD, the SWI/SNF complexes contain subunits harboring bromodomains, which recognize acetylated histones. Specifically, the SWI/SNF localizes to H3K27ac-labeled active enhancers to facilitate gene expression^58^. This can be accomplished via an increase in the accessibility to transcription factors, as the SWI/SNF complex has the ability to mobilize and shift nucleosomes^59,60^. Multiple subunits of the SWI/SNF complex have been associated with gastric cancer development^61–64^. Most notably, unbiased genome and exome sequencing studies have identified *ARID1A*, a gene encoding the DNA interacting subunit of the SWI/SNF complex^65^, as the second most frequently mutated gene after *TP53* in GC^5,66,67^. Several clinical studies have shown that *ARID1A* expression negatively correlates with increased invasion and metastasis and a worse prognosis, further supporting its role^68–70^. Cell culture studies using GC cell lines have shown that *ARID1A* loss promotes GC cell proliferation and migration^71–74^. Consistent with these data, we generated novel gastric tumor mouse models and demonstrated a tumor suppressor role of *Arid1a* in vivo^75^.

Unlike *ARID1A*, the increased expression of *BRG1*, the ATPase subunit of the SWI/SNF complex, appeared to correlate with gastric cancer development and metastasis^76^. Consistently, stabilization of BRG1 promoted EMT via the upregulation of *SNAIL*^77,78^. Interestingly, the expression of its mutually exclusive subunit, *BRM*, was lost during gastric carcinogenesis, highlighting the complex interactions between different subunits of the same chromatin remodeling complex.

**Fig. 2:**
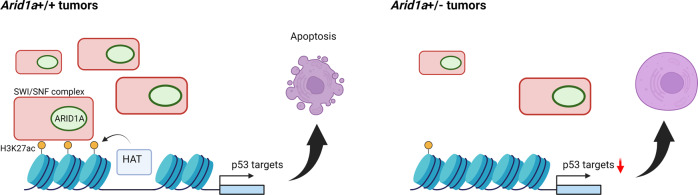
ARID1A regulation of H3K27ac in gastric tumorigenesis. The chromatin remodeling complex, SWI/SNF, is known to recognize histone acetylation via its reader subunit. The complex utilizes ATP-dependent helicase activity to slide nucleosomes and increase chromatin accessibility. This allows other histone acetyltransferases (HATs) to maintain h3K27ac, a histone modification associated with active gene expression. The loss of one copy of *Arid1a* in gastric tumors results in loss of H3K27ac at the enhancer and subsequent downregulation of p53 target genes followed by suppression of apoptosis, leading to tumor progression.

In addition to its role as a chromatin remodeling complex, the loss of SWI/SNF subunits led to the global remodeling of the H3K27ac landscape, suggesting that it can regulate H3K27ac^58,79,80^. This function of the SWI/SNF complex may be facilitated by its association with histone acetyltransferases and HDACs^81^. Alternatively, the SWI/SNF complex may increase chromatin accessibility for other HATs to deposit H3K27ac. Corroborating these data, our H3K27ac analysis of mice with the heterozygous deletion of *Arid1a* showed that the loss of the H3K27ac^+^ enhancers was associated with the p53 and apoptosis pathway genes, followed by the corresponding reduction of their expression^75^. This study demonstrates that *Arid1a* is required for the maintenance of active enhancers in genes involved in the tumor suppressor pathway, providing new mechanistic insight into the epigenetic regulation of GC (Fig. 2).

Given that the SWI/SNF and PRC2 complexes may regulate a common substrate such as H3K27, they may interact to control gene expression. Indeed, their antagonistic relationship has been demonstrated in multiple systems^82–84^. Furthermore, EZH2 inhibition has been shown to provide a specific vulnerability for *ARID1A*-mutated ovarian and gastric cancers, further supporting the interactions between writers and readers in cancer^84,85^.

#### ISWI and INO80

Much less is known about the ISWI and INO80 complexes in the context of GC. The ISWI subfamily contains a few complexes, including RSF and NURF complexes. The high expression levels of *RSF-1* (part of the RSF complex) were associated with poor prognosis in GC^86^, and knockdown of the NURF complex subunits showed tumor suppressive effects in GC cells, further supporting their roles in GC^87^. While the role of the INO80 complex in gastric carcinogenesis is relatively unexplored, it contains YY1 as a subunit^88^, a transcription factor known to activate multiple oncogenic pathways in GC^89–94^. However, whether this activation is mediated by INO80 has yet to be elucidated.

These recent studies have shown that mutations in chromatin remodeling complexes and/or their altered expression levels promote gastric carcinogenesis by abnormal activation or inhibition of oncogenic and tumor-suppressive pathways. These chromatin complexes also interact with writers and readers to establish and maintain the oncogenic histone code.

### The oncogenic chromatin landscape in gastric cancer

Since histone modification is closely tied to transcriptional regulation, it acts as a common mechanism for the dysregulation of gene expression during cancer development. The gain of active histone marks at the promoters/enhancers of oncogenes can lead to their increased expression, while the gain of repressive marks at the promoters/enhancers of tumor suppressors can lead to their reduced expression. These changes are often mediated by alterations in the expression or localization of writer proteins^6^. Here, we discuss recent studies providing new insight into how the oncogenic chromatin landscape influences GC through the regulation of promoters and enhancers.

With the advent of next-generation sequencings, such as chromatin immunoprecipitation sequencing (ChIP-seq), scientists have been able to investigate genome-wide changes in histone modification during gastric carcinogenesis. Using Nano-ChIP-seq for H3K4me1 and H3K4me3 (enhancer- and promoter-associated histone marks, respectively) and H3K27ac (active histone mark), Muratani et al. identified hundreds of H3K4me3^+^ promoters and H3K4me1^+^ enhancers whose activity differs between GC and normal tissue^95^. They defined the promoters that are gained in cancer, demonstrating that genes associated with these promoters are enriched in cancer-related gene sets and have prognostic values. This study suggested that the activation of promoters may be a critical driver of carcinogenesis. In addition, by integrating the chromatin profiles from ENCODE, the authors also found an enrichment of the PRC2 subunits EZH2 and SUZ12 in the promoters that are gained/lost in cancer, highlighting the role of PRC2 in maintaining the cancer chromatin landscape.

**Fig. 3:**
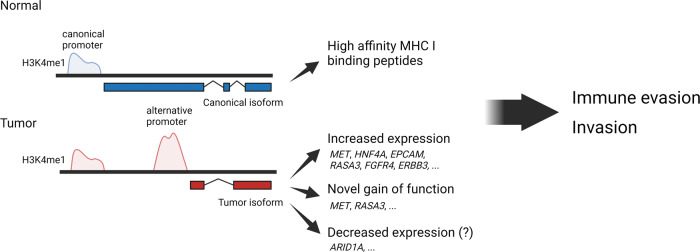
Alternative promoters in gastric carcinogenesis. Alternative promoters recognized by H3K4me are frequently identified in GC. These promoters activate the expression of tumor-specific RNA and protein isoforms. These isoforms are highly expressed in tumors. In some cases, these tumor protein isoforms can have gain of function that can promote tumor invasion (e.g., MET and RASA3). Rarely, tumor isoforms are found to be downregulated in cancer compared to control, as in the case of *ARID1A*, specifically in GC of the CIN subtype. Furthermore, the expression of these tumor isoforms can result in a lower relative expression of canonical peptides with high affinity to MHC I, leading to decreased antigen presentation and immune evasion.

Muratani et al. also identified the presence of alternative promoters in GC^95^. These promoters, not located at their canonical transcriptional start sites (TSSs), drive the expression of noncanonical transcripts and protein isoforms, which could lead to the activation of oncogenic pathways. Another Nano-ChIP-seq study by the same group further expanded on the mechanisms of alternative promoters. They found that the use of alternative promoters in GC led to an overall decrease in the expression of peptides having a strong affinity for MHC class 1 molecule, thus leading to decreased antigen presentation and potential immune evasion^96^. Recently, Sundar et al. performed single-cell RNA-seq in gastric tumors and integrated the results of a previous study to stratify the tumors based on alternate promoter usage. They found that tumors with low alternative promoter usage contain more infiltrating T cells than tumors with high alternative promoter usage, supporting the role of alternative promoters in immune evasion^97^. The presence of these alternative promoters has subsequently been confirmed across a number of cancer types in a recent large-scale RNA-seq study, suggesting that they represent one of several new mechanisms underlying dysregulated gene expression during carcinogenesis^98^. A more recent study utilized long-read RNA-seq with multiple GC cell lines and showed that noncanonical transcripts from alternative promoters may also have different coding sequences and 3’ UTRs than canonical transcripts. Furthermore, the authors found that the use of alternative promoters for specific cancer genes such as *ARID1A* is associated with different survival outcomes and molecular subtypes in GC^99^, suggesting that alternative promoters can be utilized to alter the activity of key readers involved in gastric carcinogenesis (Fig. 3).

In addition to this promoter regulation, enhancers also play a key role in GC development. Ooi et al. analyzed superenhancers, which are large enhancer regions characterized by high levels of active chromatin marks and coactivator binding. They found that superenhancers gained in GC compared to normal tissue are enriched in genes related to important cancer hallmarks, including invasion, angiogenesis, and resistance to cell death^100^. Another recent study employed paired-end ChIP-seq of H3K27ac to identify chromosomal rearrangements involving enhancers in GC. The authors identified rearrangements leading to enhancer hijacking, in which an enhancer from one region of the genome relocates to activate a gene not normally under its regulation. This led to the activation of oncogenes such as *CCNE1*^101^. These studies showed that functional elements on chromatin, as defined by histone marks, can be remodeled to cause dysregulation of gene expression in GC.

The tumor microenvironment (TME) plays an important role in providing tumors with the factors necessary to facilitate their growth and progression. The chromatin landscape of TME in gastric cancer has recently been explored. Maeda et al. examined cancer-associated fibroblasts (CAFs) in GC by performing H3K27me3 ChIP-seq and found that H3K27me3 (a repressive mark) is lost in CAFs for genes associated with the gastric stem cell niche, such as *WNT5A* when compared to normal fibroblasts^102^. In addition, a recent study found that SAA1, a secreted protein involved in the migration of GC cells, is upregulated in CAFs compared to normal fibroblasts, which may be mediated by increased H3K27ac at its enhancers and promoter^103^. These studies provide new evidence that GC development can be promoted by epigenetic changes in the tumor microenvironment. Together, genome-wide chromatin analyses of both GC cells and the TME have provided new mechanistic insight into how oncogenic and tumor suppressor pathways are epigenetically regulated in gastric tumorigenesis.

## Noncoding RNAs in gastric cancer

Over the last decade, noncoding RNAs (ncRNAs) have emerged as key players in the epigenetic regulation of carcinogenesis. ncRNAs are classified into short and long noncoding RNAs (lncRNAs), both of which regulate the expression of many key signaling pathways and regulator genes. One type of extensively studied short noncoding RNAs in GC are microRNAs (miRNAs), which are RNAs less than 30 nucleotides in length that silence complementary mRNA by either mRNA cleavage or translation repression^104^. In contrast, long noncoding RNAs (lncRNAs) are more than 200 nucleotides long, and they possess regulatory roles in facilitating miRNA–mRNA–protein crosstalk^105^. Here, we discuss recent discoveries addressing the roles of miRNAs and lncRNAs in gastric tumorigenesis.

### miRNAs regulate the components of key oncogenic pathways in gastric cancer

While expression profiling experiments previously identified differentially expressed miRNAs between GC and normal gastric cells in vitro^106^, analysis of human GC and preneoplastic lesions identified distinct miRNAs that are up- or downregulated at different stages of the disease^107^. These studies suggested that miRNAs may play key roles during each step of gastric carcinogenesis.

By regulating key oncogenic pathways such as PI3K/AKT signaling, miRNAs can act as both tumor suppressors and oncogenes^108–110^. For example, *miR137* acts as a tumor suppressor by repressing *AKT*, whereas *miR-21*, *miR-221*, and *miR-222* promote gastric tumor cell proliferation by targeting *PTEN*, a negative regulator of *AKT*^111,112^. Additional miRNAs involved in the PI3K/AKT pathway have been identified. miR-107 promoted GC cell proliferation and metastasis by targeting *FOXO1*, a transcription factor downstream of AKT signaling, and *FAT4*, a tumor suppressor suppressing PI3K/AKT signaling^113,114^. Other miRNAs, such as *miR-575* and *miR-32-5p*, were found to target *PTEN*, either directly or indirectly^115,116^. Additionally, by targeting the components of PI3K/AKT signaling, functional studies showed that *miR-20b* and *miR-451a* have opposing roles in gastric carcinogenesis^117^.

Another key oncogenic pathway regulated by miRNAs in GC is the Wnt signaling pathway, which is activated in approximately 46% of GC cases^118,119^. In a recent study, Deng et al. found significant upregulation of *miR-192* and *miR-215* in GC tissue samples and showed that these miRNAs activate Wnt signaling by targeting *APC*, a negative regulator of the pathway^120^. Another study illustrated the tumor suppressive role of *miR-520f-3p* in GC by targeting *SOX9*, an HMG-box transcription factor, and subsequently inactivating Wnt signaling^121^. These findings demonstrate that miRNAs regulate key oncogenic signaling pathways and that their expression is tightly regulated in gastric tumorigenesis.

### LncRNAs regulate gastric tumorigenesis by facilitating crosstalk between miRNAs and their target genes

By interacting with DNA, mRNA, miRNA, and histone-modifying complexes, lncRNAs can regulate transcription, post-transcription, translation, and epigenetic modifications. They play key roles in tumorigenesis and have been shown to regulate cell proliferation, the cell cycle, apoptosis and metastasis^122^. Several lncRNA expression profile studies found aberrant expression patterns of lncRNAs in GC. A comparison of lncRNA expression profiles in gastric tumor tissues to adjacent nontumorous tissues identified two differentially expressed lncRNAs, *uc001lsz* and *H19*. As the second most downregulated lncRNA in gastric cancer, *uc001lsz* was proposed to have a tumor-suppressive, trans-acting effect on *MUC2*, which is highly expressed in GC. In contrast, the authors found that *H19* was the most upregulated lncRNA in gastric tumor samples^123^. Consistent with this expression pattern, *H19* is involved in multiple processes of gastric tumorigenesis. For example, Yang et al. demonstrated that *H19* partially inactivates p53 in GC cells, while another study showed that the ectopic expression of *H19* promotes gastric tumorigenesis by acting as a precursor of miR-675^124,125^. Other studies have also demonstrated that the H19/miR-675 axis promotes GC cell proliferation by targeting the tumor suppressor RUNX1^126,127^. In addition to encoding *miR-675*, *H19* also interacts with other miRNAs to promote gastric tumorigenesis. For example, *H19* promoted tumorigenesis via the miR-138/E2F2 axis^128,129^. Moreover, the upregulation of *miR-22-3p* rescued H19-induced GC cell growth^130^.

*HOTAIR*, another lncRNA upregulated in GC, was shown to promote tumorigenesis through its interactions with miRNAs and target proteins^131–133^. Specifically, *HOTAIR* promoted proliferation by negatively regulating *miR-126* and activating CXCR4/RhoA signaling^132^ and enhanced gastric cancer cell invasion by inducing the ubiquitination of *RUNX3*, an upstream regulator of the tight junction protein CLAUDIN1^133^. Recently, *HOTAIR* has also been reported to promote gastric tumorigenesis by downregulating the exosomal levels of *miR-30a* and miR*-30b*, providing insight into the extracellular role of HOTAIR in GC^134^.

In addition to *H19* and *HOTAIR*, other lncRNAs identified from lncRNA profiling in GC are beginning to gain attention^135,136^. For example, *PVT1* has been shown to promote gastric tumorigenesis through its interactions with *FOXM1*, *miR-125*, and *miR-30a*^137–139^. Enriched in advanced GC^140^, *MALAT1* has been shown to promote GC cell proliferation by recruiting SF2/ASF, a splicing factor, downregulating *miR-204*, and activating the PI3K/AKT pathway^141–144^. In summary, these studies show that the expression levels of specific lncRNAs are tightly regulated in GC tumorigenesis and that they interact with both miRNAs and target proteins to promote cancer progression.

## Interaction between ncRNAs and histone modification in gastric cancer

**Fig. 4:**
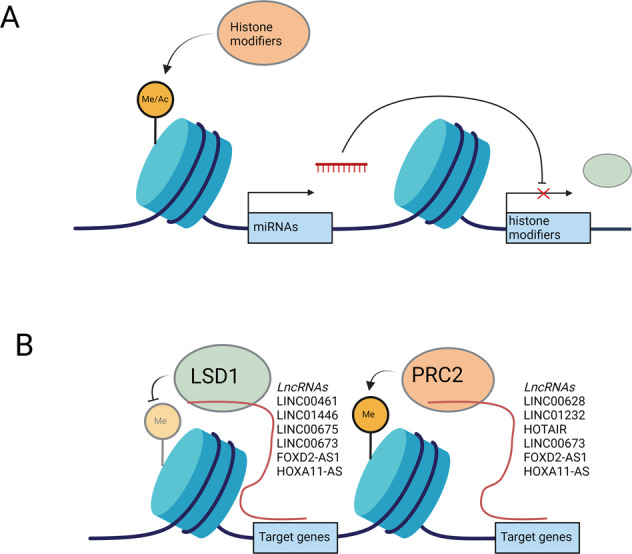
Interaction between ncRNAs and histone modification. **A** miRNAs regulate the expression of histone modifiers, which are required to maintain the chromatin landscape. miRNAs themselves are regulated by histone modifiers. miRNA and histone modifiers can become dysregulated in GC, leading to the altered expression of oncogenes and tumor suppressors. **B** LncRNAs guide histone modifiers such as PRC2 and LSD1 to their target genes. Changes in lncRNA expression in GC lead to the dysregulated expression of their target oncogenes and tumor suppressors.

The different modes of regulation in cells do not function in isolation but are interwoven in an intricate network to cooperatively regulate gene expression. As our understanding of both histone modification and ncRNAs grows, increasing interest is also focused on how their interactions control gastric tumorigenesis. Despite their distinct mechanisms of action, both miRNAs and lncRNAs influence histone modifications and are in turn regulated by these modifications in a reciprocal manner (Fig. 4).

### miRNAs and histone modifiers reciprocally regulate each other to promote or repress gastric tumorigenesis

Recent studies have examined the interactions between miRNAs and histone writers, demonstrating that they regulate each other in a reciprocal manner to promote or suppress tumorigenesis. *miR-130b-3p* promoted GC proliferation by inhibiting *MLL3a*, an H3K4 methyltransferase, in M2 macrophages^145^, while *miR-4513* stimulated both GC proliferation and EMT by targeting KAT6B^146^.

In addition to histone writers, miRNAs also interact with erasers to either suppress or promote gastric tumorigenesis. For example, the regulation of KDM by miRNAs is well characterized for its tumor-suppressing effect. *miR-194* was found to target *KDM5B*, and its overexpression suppressed GC growth both in vitro and in vivo^147^. Similarly, the upregulation of *miR-29b* suppressed gastric tumorigenesis by targeting *KDM2A*^148^. More recently, *miR-329* and *miR-491-5p* were identified as regulators of *KDM1A* and *KDM4B* (*JMJD2B*) in suppressing tumorigenesis, respectively^149,150^. In contrast, *miR-543* and *miR-204* promoted proliferation and reversed SIRT1-induced EMT in GC cells by downregulating *SIRT1*, respectively^151,152^.

Alternatively, chromatin modifiers are also capable of regulating miRNAs in GC. For example, SIRT7-mediated H3K18ac repressed *miR-34a*, leading to the inhibition of apoptosis in GC cells^153^. A recent study also showed that BRD4 represses *miR-216a-3p*, a negative regulator of *Wnt3a*, thereby promoting the stemness of GC cells through the upregulation of Wnt signaling^154^. Additionally, deletion of *HDAC1* led to an upregulation of *miR-34a*, resulting in the suppression of *miR-34a*’s downstream oncogene *CD44*, thereby inhibiting the metastasis of GC cells^155^. In contrast, deletion of *LSD1* resulted in the downregulation of *miR-142-5p*, which led to the upregulation of its tumor-suppressing target *CD9*, thereby repressing gastric cancer cell migration^43^. These results highlight the existence of a complex miRNA-histone modifier network in which miRNAs and histone modifiers reciprocally regulate each other in gastric tumorigenesis.

### lncRNAs act as guides and/or scaffolds for histone modifiers

While miRNAs typically regulate the expression of histone-modifying proteins and complexes, lncRNAs interact directly/indirectly with writers and erasers, often recruiting them to their target genes to perform histone modifications. In particular, multiple lncRNAs interact with EZH2, a subunit of PRC2, to either attenuate or promote gastric tumorigenesis. A screen for lncRNAs in GC patients identified *LINC00628* as a tumor suppressor, and *LINC00628* suppressed tumorigenesis by interacting with EZH2 and guiding PCR2 to oncogenes such as *CCNA2* and *HOX11*, leading to their methylation and silencing^156^. A more recent study suggested that lncRNAs can regulate gene expression by indirectly recruiting EZH2 through a mediator. In this study, Han et al. showed that lncRNA PART1, a lncRNA significantly downregulated in GC, upregulates *PLZF* via its interaction with androgen receptor (AR). PLZF then recruits EZH2 to increase H3K27 trimethylation at the *PDGFB* promoter, suppressing its expression and subsequently downregulating PI3K/Akt signaling^157^. Two additional lncRNAs, *LINC01232* and *LINC00202*, were found to be upregulated in GC, and they interacted with EZH2, negatively regulating the expression of *KLF2*, a zinc-finger transcription factor, through histone methylation^158,159^. Another oncogenic lncRNA, *HOTAIR*, promoted EMT by recruiting PRC2 to catalyze H3K27me3 at the *CDH1* promoter in GC^160^. Interestingly, EZH2 was recently shown to act upstream of lncRNAs. Zhu et al. demonstrated that EZH2 could methylate the promoter of a lncRNA, *PCAT18*, possibly contributing to its low expression in GC. The authors showed that *PCAT18* could inhibit GC cell proliferation by regulating *P16* expression^161^. These studies highlight the importance of ncRNA-histone writer interactions in gastric tumorigenesis.

Interactions between lncRNAs and erasers, specifically demethylases such as LSD1, also play crucial roles in gastric tumorigenesis. A recent study identified the upregulation of *LINC00461* in GC tissues and showed that its knockdown in GC cells decreases cell viability and proliferation. Further evidence indicated that *LINC00461* mediates GC cell viability via direct interaction with LSD1^162^. In light of this finding, more recent studies have uncovered the downstream mechanisms of lncRNA interactions with LSD1. For example, Lian et al. showed that *LINC01446* promotes GC cell proliferation and metastasis by epigenetically silencing *RASD1*, a member of the Ras family of monomeric G proteins, through the recruitment of LSD1 to the *RASD1* promoter^163^. In contrast, Pan et al. found that the binding of *LINC00675* to LSD1 suppresses the transcription of *SPRY4*, an upstream effector of RAS, thereby reducing GC cell proliferation and migration. Interestingly, the overexpression of *LINC00675* led to a stronger binding capacity of LSD1 to H3K4me2 and decreased H3K4me2 levels at the *SPRY4* promoter, suggesting that *LINC00675* regulates *SPRY4* expression through LSD1 in GC^164^.

Interestingly, some lncRNAs can interact with both writer and eraser, highlighting their complex mechanistic roles in gastric tumorigenesis. For example, *LINC00673* promoted GC cell proliferation by repressing key tumor suppressors, such as *KLF2* and *LATS2*, through its interaction with EZH2 and LSD1. Specifically, *LINC00673* facilitated EZH2 and LSD1 binding to the promoters of *KLF2* and *LATS2* to induce H3K27 trimethylation and H3K4 demethylation, respectively^165^. Another study found that *FOXD2-AS1* promotes GC progression by recruiting both EZH2 and LSD1 to repress *EPHB3* expression^166^. These results provide compelling evidence that lncRNAs can interact with multiple histone modifiers to regulate gastric tumorigenesis.

Recent studies have shown that lncRNAs are also able to act as scaffolds for multiple histone modifiers. For example, *GClnc1* interacted with both WDR5 and KAT2A. *GClnc1* promoted gastric tumorigenesis by acting as a scaffold for WDR5/KAT2A and recruiting them to the promoter of *SOD2*, a potential oncogene, which subsequently facilitated its expression by increasing H3K4me3^167^. Another study showed that *GCAWKR* recruits WDR5/KAT2A to upregulate the oncogene *PTP4A1*^168^. In addition to *GClnc1* and *GCAWKR*, *HOXA11-AS* can also act as a scaffold for both histone modifiers and DNA methyltransferases, which include EZH2, LSD1, and DNMT1. Specifically, *HOXA11-AS* interacts with DNMT1/EZH2 to repress *KLF2* expression through H3K27me3, while its interaction with LSD1/EZH2 represses *PRSS8* expression through H3K27me3 and H3K4 demethylation^169^.

Finally, new studies have begun to explore lncRNA interactions with readers. For example, lncRNA *NEAT1* directly interacted with BRG1, a subunit of the SWI/SNF complex, which resulted in the silencing of the G2/M checkpoint protein *GADD45A* and subsequently promoted GC cell proliferation^170^. In summary, these studies provide valuable insight into the diverse roles of lncRNA-histone modifier interactions, revealing a complex epigenetic network involved in the regulation of gastric tumorigenesis (Fig. 4).

## Concluding remarks

The three main types of epigenetic regulation include DNA methylation, histone modification, and ncRNAs. While changes in DNA methylation remain one of the major mechanisms for gastric carcinogenesis, recent studies have shown that histone modifications and ncRNA also play critical roles. In this review, we discuss recent findings addressing the mechanism of histone modification and ncRNAs in GC. Specific writer and eraser proteins control histone modification and interact with readers such as chromatin remodeling complexes. Their dysregulation in GC leads to altered expression levels of downstream oncogenes and tumor suppressor genes. In addition to histone modification, ncRNAs regulate many key signaling pathways and genes involved in the development of GC. Importantly, these histone modifications and ncRNAs also interact with and regulate each other.

While somatic mutations of oncogenes and tumor suppressors are thought to be the major cause of cancer, accumulating evidence has increasingly supported the importance of epigenetic regulation in carcinogenesis. Since certain environmental factors are known to increase the risk of GC, epigenetic regulation would likely serve as a critical link between the environment and gene expression. Of note, the stomach is constantly exposed to environmental factors. Infection with two gastric pathogens, Epstein‒Barr virus (EBV) and *H. pylori*, is known to increase the risk of GC^11,13^. EBV in particular is known to cause widespread DNA methylation in GC, suggesting that repression of tumor suppressors by promoter hypermethylation is a core mechanism in EBV-mediated carcinogenesis^15^. Notably, a few recent studies have begun to explore the global changes in the chromatin landscape caused by EBV infection. Hi-C analysis revealed physical interactions between the EBV genome and the host genome, demonstrating EBV-mediated global changes in histone modification^171^. Similar to EBV-mediated DNA methylation, *H. pylori* can influence the expression of key regulators through altered DNA methylation^172^. It is also known to cause dysregulation of writers, readers, miRNAs, and ncRNAs, highlighting its potential in modifying the epigenetic landscape globally^173–180^. However, future studies are required to further define the mechanistic interactions between these environmental factors and epigenetic regulators.

New technologies continue to facilitate discoveries that will improve our understanding and treatment of GC. For example, combinatorial drug therapies prove to be a promising personalized medicine approach with the potential to reduce off-target toxicity^181^. In recent years, in vitro studies combining epigenetic inhibitors with other anticancer compounds have shown synergistic effects in tumor suppression for GC^75,182–184^. However, clinical trials investigating combinatorial treatment involving epigenetic inhibitors in GC have been limited^185^. In addition, novel single-cell technologies such as single-cell ATAC-seq allows us to probe the role of chromatin architecture and ncRNAs in tumor heterogeneity, which poses another major challenge in cancer treatment. Therefore, efforts to elucidate the epigenetic mechanism underlying gastric carcinogenesis will continue to improve the treatment and prevention of GC.
